# Phenotypic Analysis of Intentionally Created Monocular Visual Field Defects During Bilateral Randomized Visual Field Testing Using the Imo Vifa^®^

**DOI:** 10.3390/jcm15010009

**Published:** 2025-12-19

**Authors:** Yuiko Kawaguchi, Yuki Takagi, Takashi Kojima, Akeno Tamaoki, Tatsushi Kaga

**Affiliations:** 1 Department of Ophthalmology, Japan Community Healthcare Organization Chukyo Hospital, 1-1-10 Sanjo, Minami-ku, Nagoya 457-8510, Aichi, Japan; ytakagi@sanjogroup.jp (Y.T.); kojima@sanjogroup.jp (T.K.); tamaoki@chukyogroup.jp (A.T.); kaga@sanjogroup.jp (T.K.); 2 Department of Ophthalmology, Japan Community Healthcare Organization Kanitono Hospital, 1221-5 Dota, Kani 509-0206, Gifu, Japan; 3 Nagoya Eye Clinic, Kanayama, COLLECT-MARK, 24-14, Namiyose-cho, Atsuta-ku, Nagoya 456-0003, Aichi, Japan

**Keywords:** visual field test, imo Vifa^®^, bilateral randomized visual field testing, simulated visual field defect

## Abstract

**Background/Objectives:** The imo Vifa^®^ is reportedly useful for diagnosing functional visual field loss; however, its potential for detecting malingering is unclear. Here, we intentionally simulated monocular visual field defects under bilateral randomized visual field testing conditions using the imo Vifa^®^ in healthy participants and compared their resulting defect phenotypes. **Methods:** Twenty participants (mean age, 37.3 ± 12.4 years; 12 orthoptists, 1 physician, and 7 administrative staff members) without ocular disease were enrolled. Four types of monocular visual field defects were simulated: right eye nasal hemianopia, left eye temporal hemianopia, right eye centripetal visual field constriction, and left eye central scotoma. Bilateral randomized visual field testing was performed using the AIZE-rapid mode with the 24-2 and 24plus(1) programs. **Results:** Accurate simulation of the intended defects was challenging. Orthoptists produced left homonymous hemianopia for right nasal hemianopia and left temporal hemianopia. Regarding right nasal hemianopia, many office workers generated patterns resembling right homonymous hemianopia-like, whereas for left temporal hemianopia, noncertified orthoptists produced patterns similar to those of left homonymous hemianopia-like. Considering the right centripetal constriction, all orthoptists produced the intended centripetal constriction, whereas non-orthoptists generated right homonymous hemianopia-like or patchy patterns. Orthoptists produced central scotomas or patchy patterns for the left central scotoma, whereas non-orthoptists generated left homonymous hemianopia-like patterns. **Conclusions:** Creating targeted monocular abnormalities during bilateral randomized visual field testing was challenging. Differences in the participants’ understanding of visual field testing influenced the resulting patterns. In future research, having participants create monocular visual field defects under occlusion conditions would be necessary.

## 1. Introduction

Functional visual loss refers to visual impairment, including decreased visual acuity or visual field defects in the absence of any organic ocular pathology [1]. This impairment is most frequently observed in girls aged 9–11 years [2]. Meanwhile, malingering is the deliberate feigning of visual loss, often for secondary gains such as economic or legal benefits [3]. However, visual evoked potentials can be objectively measured and are considered useful for diagnosing psychogenic visual field defects [4,5,6]. Ruling out organic disorders, it can be demonstrated that visual function is not impaired [4].

Patients with functional visual field defects often exhibit concentric constrictions, spiral-shaped fields, or irregular and inconsistent patterns [7]. Binasal hemianopia is rare [4,8]. Goldmann perimetry (GP) has traditionally been considered one of the most useful methods for diagnosing functional visual field defects [3,9]. However, in cases wherein GP cannot establish a diagnosis, testing using a Humphrey Field Analyzer (HFA) might be more effective [8]. Additionally, repeating GP at varying test distances can assist in differentiating between non-organic defects [10].

Since both GP and HFA require monocular testing, individuals are aware of which eye is being examined. In contrast, the imo Vifa^®^ perimeter (CREWT Medical Systems, Tokyo, Japan) enables bilateral randomized visual field testing. Using the imo Vifa^®^, stimuli are presented separately to each eye on independent displays, allowing for simultaneous or monocular measurements under binocular conditions. Previous studies have also reported that the imo findings strongly correlated with those on the HFA [11,12] and the examination time is significantly shorter using the imo Vifa^®^ [12].

However, since the imo Vifa^®^ uses a unique stimulus sequence, direct comparison with the HFA is not always possible [13]. Since the imo Vifa^®^ allows binocular viewing and individuals cannot discern which eye is being tested [1,12,14,15], it may be more difficult for malingering patients to manipulate their responses intentionally.

The imo Vifa^®^ has been reported to be useful for diagnosing functional visual field loss [1,14]. Takagi et al. demonstrated that repeated bilateral randomized visual field testing using the imo Vifa^®^ revealed inconsistencies in patients with monocular functional visual loss [1]. This approach may be useful for diagnosing patients with psychogenic monocular visual field defects. However, to date, no studies have assessed the potential of the imo Vifa^®^ for detecting malingering. If healthy individuals attempt to simulate malingering, the resulting visual field patterns could provide valuable insights into the tendencies of feigned defects.

The imo Vifa^®^ features a unique testing program termed 24plus(1). The 24-2 program includes 54 test points, whereas the 24plus(1) uses only 36 points arranged in an original imo-specific pattern that focuses on regions where visual field defects commonly occur [12].

If 24plus(1) yields results comparable to those of 24-2, its shorter testing time might make examining patients with suspected functional or malingering visual loss particularly useful. In this study, we aimed to evaluate whether healthy individuals could intentionally simulate monocular visual field defects under bilateral randomized visual field testing conditions using the imo Vifa^®^. We compared the results of the 24-2 and 24plus(1) programs and analyzed the characteristic trends in the simulated field patterns.

## 2. Materials and Methods

### 2.1. Participants

The prospective study included 20 healthy participants (40 eyes) without any history of ocular disease, recruited from the Department of Ophthalmology at Chukyo Hospital. The cohort comprised 13 orthoptists (ORTs), 1 physician, 6 administrative staff members, and 1 ORT trainee, all of whom provided consent prior to participation. The mean age of the participants was 37.3 ± 12.4 years. None of the selected administrative staff members had prior experience with visual field testing. No exclusion criteria were established.

This study adhered to the tenets of the Declaration of Helsinki and was approved by the Institutional Ethics Committee of Chukyo Hospital (approval no.: 2023044; 20 November 2023).

### 2.2. Experimental Design

**Figure 1:**
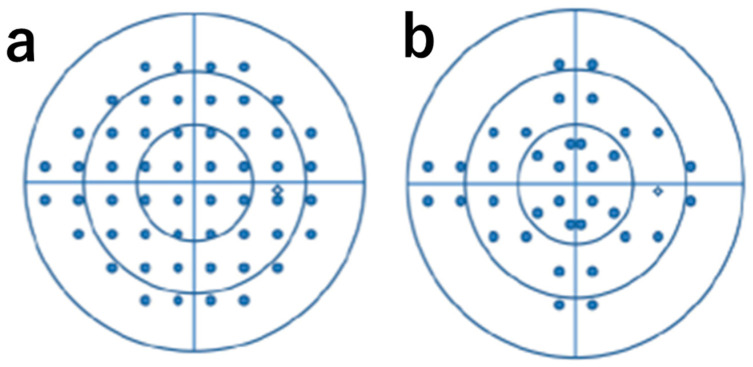
**24-2 and 24plus(1) test location**: (**a**) arrangement of measurement points for 24-2 and (**b**) arrangement of measurement points for 24plus(1).

The participants were instructed to deliberately simulate four types of monocular visual field defects: right nasal hemianopia, left temporal hemianopia, right concentric constriction, and left central scotoma, during bilateral randomized visual field testing using the imo Vifa^®^. Each participant underwent both the 24-2 and 24plus(1) test modes using Ambient Interactive Zippy Estimation (AIZE)-rapid; the order of the simulated defect types and test modes was randomized (Figure 1).

### 2.3. Data Analysis

We calculated the visual field creation rate, defined as the percentage of tests during which the participants successfully created the intended monocular defect. We also determined the consistency rate, defined as the percentage of identical visual field defect patterns between the 24-2 and 24plus(1) programs. The visual fields were classified based on grayscale patterns.

Statistical analyses were performed using IBM SPSS Statistics version 21 (IBM Corp., Chicago, IL, USA). Normality of data distribution was assessed using the Shapiro–Wilk test; paired *t*-tests were used to compare test durations between the 24-2 and 24plus(1). Statistical significance was set to *p* < 0.05.

## 3. Results

Regarding all testing modes, none of the participants were able to intentionally create monocular visual field defects under bilateral randomized visual field testing conditions across the four instructed patterns. Therefore, none of the simulated patterns matched the instructed monocular pattern under our predefined criteria.

The concordance rates of the simulated abnormal visual fields between the 24-2 and 24plus(1) modes were 70%, 95%, 75%, and 60% for right nasal hemianopia, left temporal hemianopia, right concentric visual field constriction, and left central scotoma, respectively (Table 1).

### 3.1. Right Eye Nasal Hemianopia

**Figure 2:**
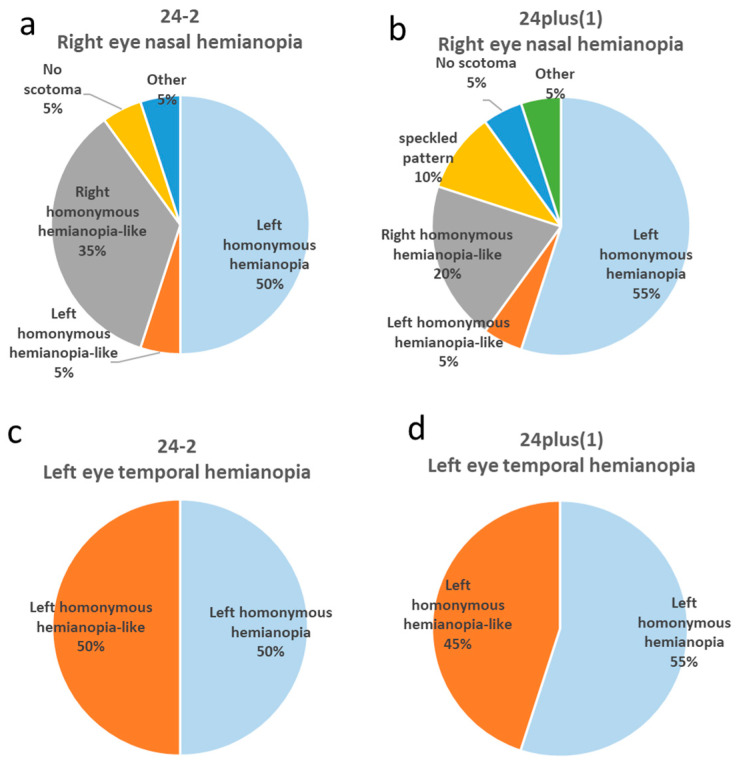
**Distribution of phenotypic patterns in right nasal hemianopia and left temporal hemianopia created under 24-2 and 24plus(1) testing modes**. (**a**) Phenotypic distribution of intentionally created right nasal hemianopia measured by 24-2. The most frequent pattern was left homonymous hemianopia (50%), followed by right homonymous hemianopia-like (35%). (**b**) Phenotypic distribution of intentionally created right nasal hemianopia measured by 24plus(1). The most frequent pattern was left homonymous hemianopia (55%), followed by right homonymous hemianopia-like (20%). (**c**) Phenotypic distribution of intentionally created left temporal hemianopia measured by 24-2. Both left homonymous hemianopia and left homonymous hemianopia-like patterns accounted for 50% each. (**d**) Phenotypic distribution of intentionally created left temporal hemianopia measured by 24plus(1). The most frequent pattern was left homonymous hemianopia (55%), followed by left homonymous hemianopia-like (45%).

Using the 24-2 program, 50% of the participants (all ORTs) showed left homonymous hemianopia; 5% (ORTs) showed left homonymous hemianopia-like patterns; 35% (administrative staff and ORT trainees) showed right homonymous hemianopia-like defects; 5% (an administrative staff member) showed no scotoma; and 5% (a physician) showed other atypical results (Figure 2a).

**Figure 3:**
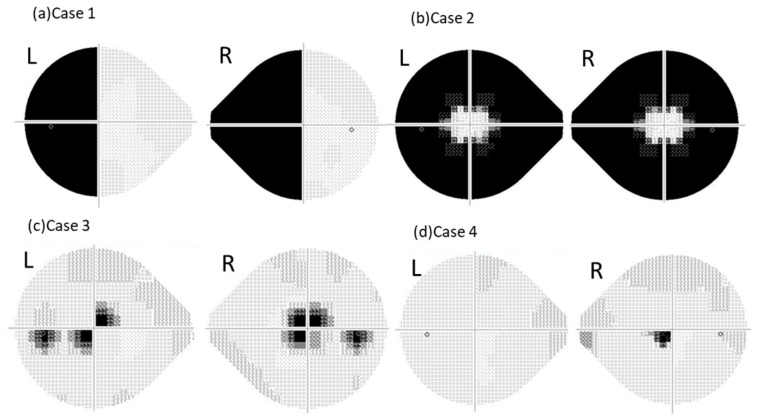
**Representative grayscale visual fields intentionally created by certified orthoptists (ORTs)**. This figure shows examples of abnormal visual fields intentionally created by the ORTs. (**a**) Case 1: The results of right nasal hemianopia instruction measured by 24plus(1), showing a left homonymous hemianopia pattern. (**b**) Case 2: The results of right centripetal visual field constriction instruction measured by the 24plus(1), showing a symmetrical centripetal constriction pattern. (**c**) Case 3: The results of left central scotoma instruction measured by 24-2, showing an asymmetrical central scotoma pattern. (**d**) Case 4: The results of left central scotoma instruction measured by 24plus(1), showing an atypical right central scotoma pattern.

**Figure 4:**
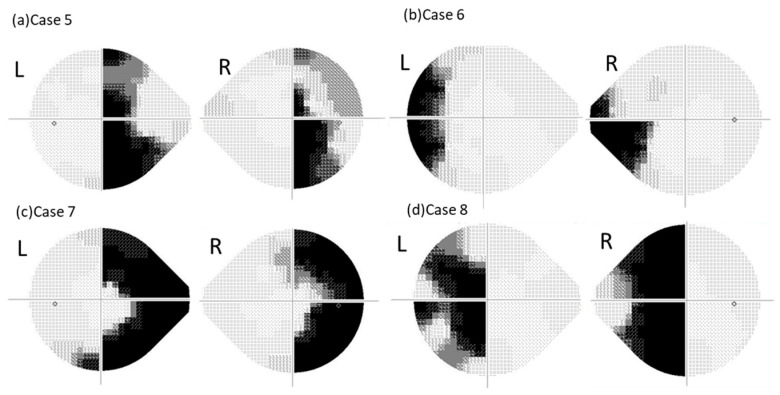
**Representative grayscale visual fields intentionally created by administrative staff and an orthoptist trainee**. This figure presents examples of abnormal visual fields intentionally created by the administrative staff and an orthoptist trainee. (**a**) Case 5: The results of right nasal hemianopia instruction measured by 24plus(1), showing a right homonymous hemianopia-like pattern (administrative staff member). (**b**) Case 6: The results of left temporal hemianopia instruction measured by 24plus(1), showing a left homonymous hemianopia-like pattern (administrative staff member). (**c**) Case 7: The results of right centripetal visual field constriction instruction measured by 24plus(1), showing a right homonymous hemianopia-like pattern (orthoptist trainee). (**d**) Case 8: The results of the left central scotoma instruction measured by 24plus(1), showing a left homonymous hemianopia-like pattern (administrative staff member).

In the 24plus(1) mode, 55% (all ORTs) exhibited left homonymous hemianopia (Figure 3a); 5% (ORTs) showed left homonymous hemianopia-like defects; 20% (all administrative staff) exhibited right homonymous hemianopia-like defects (Figure 4a); 10% (administrative staff) showed mottled patterns; 5% (the physician) showed no scotoma; and 5% (ORT trainee) showed other atypical fields (Figure 2b).

### 3.2. Left Eye Temporal Hemianopia

Using the 24-2 mode, 50% (all ORTs) produced left homonymous hemianopia; the remaining 50% (one physician, one ORT, one administrative staff member, and one ORT trainee) produced left homonymous hemianopia-like fields (Figure 2c).

Using 24plus(1) mode, 55% (all ORTs) produced left homonymous hemianopia, and 45% (one physician, one ORT, one administrative staff member, and one ORT trainee) produced left homonymous hemianopia-like results (Figure 4b; Figure 2d).

### 3.3. Right Eye Concentric Visual Field Constriction

**Figure 5:**
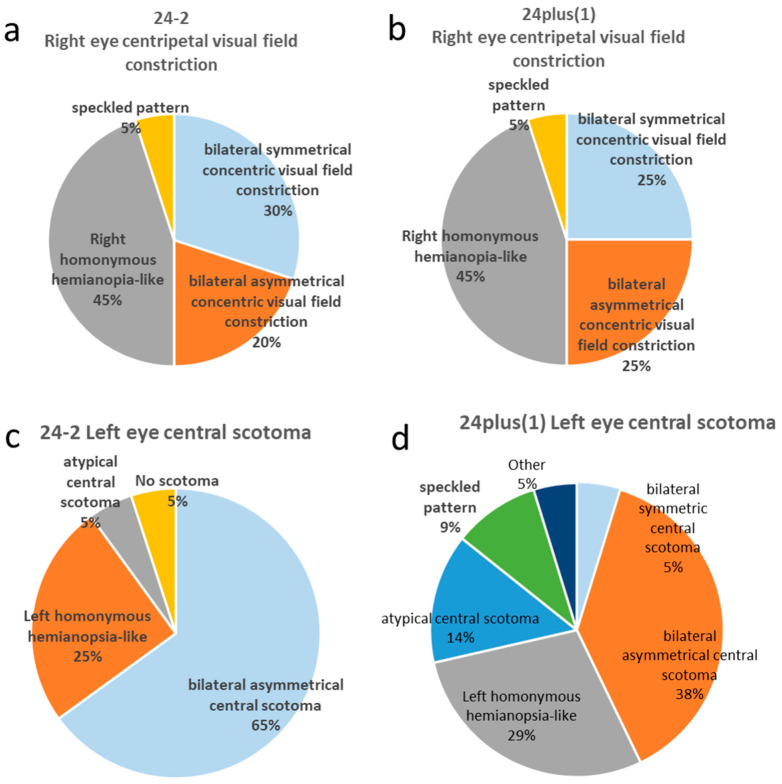
**Distribution of phenotypic patterns in right centripetal visual field constriction and left central scotoma under 24-2 and 24plus(1) testing modes**. (**a**) Phenotypic distribution of intentionally created right centripetal visual field constriction measured by 24-2. The most frequent pattern was right homonymous hemianopia (45%), followed by symmetrical (30%) and asymmetrical (20%) centripetal constriction. (**b**) Phenotypic distribution of intentionally created right centripetal visual field constriction measured by 24plus(1). The most frequent pattern was right homonymous hemianopia-like (45%), followed by symmetrical and asymmetrical centripetal constriction (25% each). (**c**) Phenotypic distribution of intentionally created left central scotoma measured by 24-2. The most frequent pattern was asymmetrical central scotoma (65%), followed by left homonymous hemianopia-like (25%). (**d**) Phenotypic distribution of intentionally created left central scotoma measured by 24plus(1). The most frequent pattern was asymmetrical central scotoma (38%), followed by left homonymous hemianopia-like (29%).

Using the 24-2, 30% (all ORTs) produced bilaterally symmetric concentric constrictions; 20% (all ORTs) showed asymmetric constrictions; 45% (one physician, one ORT, administrative staff members, and one ORT trainee) produced right homonymous hemianopia-like fields; and 5% (ORT) showed mottled patterns (Figure 5a).

Using 24plus(1), 25% (all ORTs) produced bilaterally symmetric constrictions (Figure 3b), 25% (all ORTs) produced asymmetric constrictions; 45% (one physician, one ORT, administrative staff members, and one ORT trainee) produced right homonymous hemianopia-like fields (Figure 4c); and 5% (ORT) produced mottled fields (Figure 5b).

### 3.4. Left Eye Central Scotoma

Using the 24-2 mode, 65% (ORTs and the physician) exhibited bilaterally asymmetric central scotomas (Figure 3c), 25% (administrative staff members and ORT trainees) showed left homonymous hemianopia-like fields, 5% (administrative staff) showed atypical central scotomas, and 5% (administrative staff) showed no scotomas (Figure 5c).

Using 24plus(1), 35% (all ORTs) produced bilaterally asymmetric central scotomas; 30% (administrative staff members and ORT trainees) exhibited left homonymous hemianopia-like defects (Figure 4d); 15% (ORTs and the physician) showed atypical right-eye central scotomas (Figure 3d); 10% (ORTs) showed mottled fields, 5% (ORT) produced bilaterally symmetric central scotomas; and 5% (administrative staff members) showed other patterns (Figure 5d).

### 3.5. Test Duration

**Figure 6:**
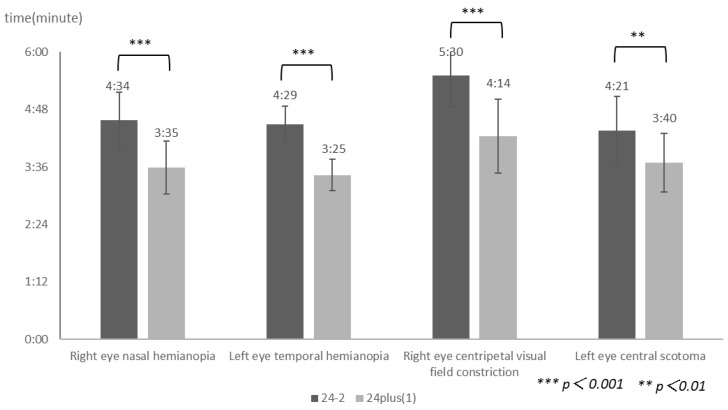
**Comparison of mean testing times between the 24-2 and 24plus(1) modes**. This figure shows the mean measurement times for right nasal hemianopia, left temporal hemianopia, right centripetal visual field constriction, and left central scotoma under the 24-2 and 24plus(1) testing modes. Regarding all types of visual field abnormalities, the testing time for 24plus(1) was significantly shorter than that for the 24-2 (*p* < 0.01).

As shown in Figure 6, the mean test durations were 274 ± 35 s, 269 ± 22 s, 330 ± 39 s, and 261 ± 42 s for right eye nasal hemianopia, left eye temporal hemianopia, right eye concentric visual field constriction, and left eye central scotoma, respectively, when measured using the 24-2 program. In contrast, the corresponding durations measured using 24plus(1) were 215 ± 33 s, 205 ± 19 s, 254 ± 46 s, and 220 ± 36 s. Across all abnormal visual field patterns, the test durations were significantly shorter using 24plus(1) compared with the 24-2 program (*p* < 0.01).

## 4. Discussion

### 4.1. Rate of Successful Simulation of Monocular Visual Field Defects

None of the simulated patterns matched the instructed monocular pattern under our predefined criteria. Since the imo Vifa^®^ presents light stimuli separately to each eye based on its characteristics, accurately determining which eye was being stimulated was challenging [1,12,14,15]. These findings suggest that the stimulus presentation characteristics of the imo Vifa^®^ might make performing simulated visual field tests difficult.

Consequently, the success rate of generating abnormal monocular visual fields was 0%. Furthermore, even after eight test sessions in this study, physicians and ORTs with sufficient knowledge of perimetry were unable to identify which eye received the light stimulus.

### 4.2. Agreement Rate of Created Visual Fields

The 24plus(1) mode exhibited greater variability in the phenotypes of the created visual fields compared with the 24-2 mode. This is possibly attributable to the fact that, unlike the 24-2, the arrangement of the test points within the central 10° in the 24plus(1) is not organized in a grid pattern. In cases of concentric visual field constriction or central scotoma, the visual field is perceived as a circle; depending on its size, the circular area of attention might differently overlap with the test point arrangements of the 24-2 and 24plus(1), resulting in lower concordance rates. Furthermore, hemianopia demonstrated higher concordance rates compared with concentric constriction or central scotomas. Regarding hemianopia, the participants primarily focused on the vertical meridian passing through the fixation point, making the test point arrangement less influential.

However, there was a discrepancy in the concordance between nasal and temporal hemianopia. Two clerical staff members who created mottled patterns during the 24plus(1) test for nasal hemianopia underwent their first-ever visual field examination; on subsequent 24-2 tests, they produced right homonymous hemianopia-like fields. This suggests that the improved agreement in temporal hemianopia might be due to the practice effect.

Furthermore, the agreement rate for central scotoma was lower than that for concentric constrictions. This difference cannot be explained solely by knowledge or experience; rather, it possibly results from differences in the test point distribution between 24plus(1) and 24-2. Two ORTs produced atypical right eye central scotomas only using 24plus(1). In these cases, the grayscale map revealed a single depressed point immediately inferior and nasal to the fixation target (Figure 3d). In the other individuals, results similar to those of many ORTs were observed, except for the central scotoma.

In both cases, asymmetric central scotomas were seen using the 24-2 but not using 24plus(1). Since 24plus(1) has a different spatial arrangement of test points within the central 10°, a smaller consciously perceived circular area could lead to more localized scotomas compared with the 24-2. Since determining which eye could perceive a light stimulus under binocular open-view testing was difficult [15,16], atypical right-eye scotomas observed with 24plus(1) are thought to result from differences in the size of the perceived circular area, rather than from the test configuration or knowledge. Consequently, these factors might explain why the central scotomas showed lower agreement rates compared with concentric constrictions.

### 4.3. Patterns of Created Visual Fields

Simulated abnormal visual fields were broadly divided into two types. Across all test patterns, the phenotypes of the created fields tended to differ between the ORTs and other participants. Administrative staff members and ORT trainees generally produced right homonymous hemianopia-like fields when instructed to simulate right nasal hemianopia and left homonymous hemianopia-like fields for left temporal hemianopia (Figure 4a,b).

Previous reports [1,7] have shown that participants tend to exhibit hemianopia in the non-seeing eye during bilateral randomized visual field testing. Participants without prior knowledge or experience in perimetry might have assumed that the right half of the fixation point corresponded to the right eye and the left half corresponded to the left eye, resulting in patterns similar to those described in earlier studies.

The participants were required to conceptualize the visual field as a circle when creating a right concentric constriction or left central scotoma. However, administrative staff members and ORT trainees produced right homonymous hemianopia-like fields when simulating a right concentric constriction (Figure 4c). This possibly occurred because the right side of the fixation point was divided vertically, with participants perceiving the outermost area as the temporal side of the right eye and the inner area as the nasal side. Thus, they visualized the constricted field as a semicircle extending right from the fixation point, resulting in a homonymous hemianopia-like appearance. Similarly, when simulating a left central scotoma, the participants produced left homonymous hemianopia-like fields (Figure 4d).

Among these misperceptions, in cases of right-eye centripetal visual field constriction, the pattern resembled right homonymous hemianopia, with the scotoma area predominantly located on the temporal side. In cases of left-eye central scotoma, the pattern resembled a left homonymous hemianopia-like pattern, with the scotoma predominantly located slightly more centrally. One administrative staff member produced a “no scotoma” result, which might reflect difficulty in consciously generating a scotoma without prior testing experience. Of note, this participant could create clearer abnormal fields in subsequent trials, suggesting a learning effect.

In the case of ORTs who had experience with visual field testing and knowledge about visual fields, many correctly produced right homonymous hemianopia when simulating right nasal hemianopia. In other words, they interpreted the right half of the visual field from the fixation point as temporal and the left half as nasal, demonstrating that visual field testing assessed binocular single vision.

Nevertheless, some ORTs and a physician, despite having relevant experience, produced abnormal fields similar to those created by the administrative staff. One ORT (A) produced staff-like results across all conditions, another (B) only in left temporal hemianopia (24-2), and a third (C) showed atypical results in right concentric constriction (24-2). Only ORT (C) had less than one year of experience. ORTs (B) and (C) demonstrated an improved understanding of binocular testing in subsequent trials, whereas ORT (A) did not recognize this concept, even at the end of the study. These findings suggest that even trained personnel might reproduce novice-like patterns during their initial testing.

In summary, the phenotype of intentionally created visual field defects tended to differ depending on the participants’ knowledge and experience. However, experience and knowledge alone did not guarantee the typical results. As noted in the agreement rate analysis, it has been demonstrated that even individuals lacking experience and knowledge can achieve a change in their field of vision through a certain degree of practice.

### 4.4. Test Duration

The 24plus(1) mode required significantly less time than did the 24-2 (Figure 6).

This difference could be attributed not only to the distinct test point arrangements but also to the smaller number of points in 24plus(1). Despite the altered spatial distribution, the reduced test load resulted in a shorter completion time.

### 4.5. Limitation

The study has some limitations. Future studies should evaluate the difficulty in intentionally creating the four types of visual field defects under monocular occlusion using the HFA. Being unable to compare with the HFA, which allows monocular visual field testing, is a critical issue.

Furthermore, additional experiments should assess the patterns produced by the participants who previously created incorrect visual fields when tested monocularly using the imo Vifa^®^.

Some participants without prior knowledge or experience exhibited changes in the patterns of their simulated visual fields due to a learning effect. Further studies are warranted to determine whether these learning effects would occur in all participants.

In this study, the participants intentionally simulated malingering, and no comparison was made with psychogenic visual field defects. Since such differences might have influenced the outcomes, further investigation is warranted.

## 5. Conclusions

Under bilateral randomized visual field testing conditions, intentionally creating abnormal visual fields in only one eye is difficult.

Since the arrangement of test points within the central 10° differs between 24plus(1) and 24-2, variations in visual field results might occur in cases of concentric constriction or central scotoma, depending on the test point distribution.

The phenotypes of intentionally created visual fields varied depending on the level of knowledge and experience.

These results indicated that the device might have potential utility for evaluating patients with suspected malingering; however, further studies including clinical cases are warranted.

## Figures and Tables

**Table 1:** Target Field of View Creation Rate And Creation Field of View Match Rate.

	Target Field of View Creation Rate	Creation Field of View Match Rate
Right eye nasal hemianopia	0%	70%
Left eye temporal hemianopia	0%	95%
Right eye centripetal visual field constriction	0%	75%
Left eye central scotoma	0%	60%

## Data Availability

The data presented in this study are available on request from the corresponding author due to privacy.

## Abbreviations

The following abbreviations are used in this manuscript:

HFA	Humphery Field Analyzer
GP	Goldmann Perimetry
ORT	Orthoptists
